# A Pilot Study of Primary Ciliary Dyskinesia: Sleep-Related Disorders and Neuropsychiatric Comorbidities

**DOI:** 10.3390/jcm14041353

**Published:** 2025-02-18

**Authors:** Roberto A. Cardona-Quiñones, Edicer Ramírez-Rivera, Edwin Álvarez-Torres, Saidy A. Salem-Hernández, Noel J. Vargas-Pérez, Wilfredo De Jesús-Rojas

**Affiliations:** 1 Department of Psychiatry, School of Medicine, University of Puerto Rico, Medical Science Campus, P.O. Box 365067, San Juan, PR 00936-5067, USA; edicer.ramirez@upr.edu (E.R.-R.); edwin.alvarez@upr.edu (E.Á.-T.); saidysalem91@gmail.com (S.A.S.-H.); 2 Department of Neurology, School of Medicine, University of Puerto Rico, Medical Science Campus, P.O. Box 365067, San Juan, PR 00936-5067, USA; vargas.noelj@gmail.com; 3 Basic Science & Pediatrics, Ponce Health Science University, P.O. Box 7004, Ponce, PR 00732, USA; wdejesus@psm.edu

**Keywords:** primary ciliary dyskinesia, obstructive sleep apnea, sleep-related movement disorders, neuropsychiatric symptoms

## Abstract

Sleep disorders are characterized by impaired quality, timing, and amount of sleep, resulting in daytime distress and functioning. Primary ciliary dyskinesia (PCD) is a rare genetic condition characterized by oto-sino-pulmonary manifestations with multiple comorbidities, including sleep disorders. **Background/Objectives**: This pilot study aims to assess sleep disorders and neuropsychiatric comorbidities in Puerto Rican patients with the *RSPH4A* (c.921+3_921+6delAAGT) PCD founder mutation. However, the literature on sleep-related disorders and their neuropsychiatric comorbidities in PCD is limited. **Methods**: A cohort of fifteen patients with the *RSPH4A* (c.921+3_921+6delAAGT) founder mutation (six pediatric, nine adults) were evaluated for sleep quality, cognitive, neurodevelopmental history, and mood-related manifestations, followed by diagnostic polysomnography for sleep-disordered breathing and other sleep-related disorder detection. **Results**: Twelve out of fifteen (12/15, 80%) patients presented with sleep-related disorders, particularly obstructive sleep apnea where the median Pediatric AHI was 1.25/h (IQR: 1.1–1.75/h), T < 90: 0.1 min (IQR: 0–1.9 min) and adult AHI 1.3 (IQR: 0.9–8), T < 90: 0.2 min (IQR: 0–3.5 min). PCD patients also presented complex sleep behaviors, and more than half had sleep-related movement manifestations such as sleep-related Bruxism, PLMS, among others. All pediatric patients with OSA met criteria for an anxiety disorder, with a GAD-7 of 13 (IQR: 10.5–15.8); this association was not clearly seen in adults. **Conclusions**: Patients with PCD *RSPH4A* exhibited multiple sleep and neuropsychiatric manifestations, particularly OSA, sleep-related movement disorders and complex sleep behaviors. Further studies are needed to determine if these manifestations result from obstructive breathing, sleep mechanism disruption, or other neurodevelopmental impairment associated with this ciliopathy.

## 1. Introduction

Primary ciliary dyskinesia (PCD) is a rare, genetically heterogeneous disorder characterized by dysfunctional motile cilia, resulting in impaired mucociliary clearance and a range of oto-sino-pulmonary manifestations [1,2,3]. Beyond these established respiratory complications, there is growing interest in the impact of sleep-related disorders and associated neuropsychiatric manifestations within the PCD population [4,5,6]. However, the literature on sleep disturbances and their neuropsychiatric comorbidities in PCD remains sparse, particularly among Hispanic patients [7,8,9].

Notably, one of the genetic factors underlying PCD in Puerto Rico is the *RSPH4A* (c.921+3_921+6delAAGT) founder mutation [8]. Previous research has linked this genetic variant to common PCD-related complications, such as chronic respiratory infections, sinusitis, bronchiectasis, and infertility [9,10]. Yet, how this specific genetic variant may influence sleep-related disorders and other neuropsychiatric comorbidities remains poorly understood [11,12,13]. This pilot study aims to address this knowledge gap by examining the presence and characteristics of sleep disorders and neuropsychiatric comorbidities among patients with the *RSPH4A* founder mutation in Puerto Rico.

We hypothesize that patients with PCD carrying the *RSPH4A* founder mutation may exhibit a high prevalence of sleep-related disorders and neuropsychiatric comorbidities, particularly obstructive sleep apnea (OSA), sleep-related movement disorders, and anxiety or depressive symptoms. Furthermore, we propose that these manifestations may be associated with mechanisms such as obstructive breathing, altered sleep architecture, or neurodevelopmental impairment related to ciliary dysfunction.

By focusing on a genetically defined subgroup, we seek to expand the understanding of PCD beyond its classic respiratory phenotypes and explore new dimensions that may significantly affect patient’s quality of life.

## 2. Materials and Methods

### 2.1. Patients

**Figure 1:**
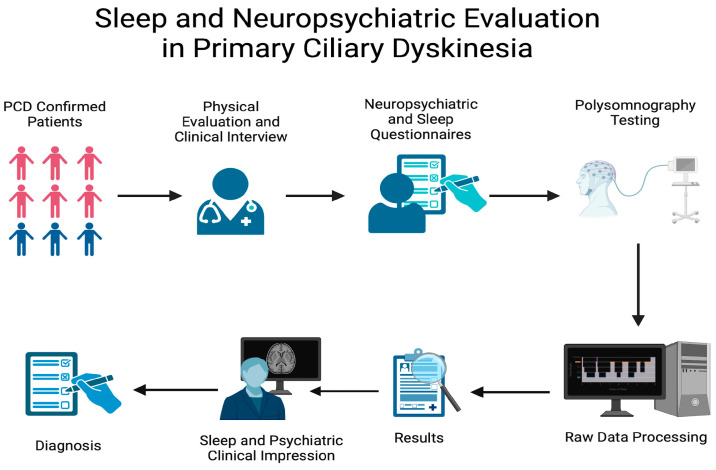
Workflow for sleep and neuropsychiatric evaluation in primary ciliary dyskinesia (PCD). The process begins with PCD-confirmed patients undergoing a comprehensive physical evaluation and clinical interview. Neuropsychiatric and sleep questionnaires are administered to assess symptoms, followed by polysomnography testing for detailed sleep analysis. Data from the polysomnography are processed to generate raw results, which are reviewed alongside clinical impressions to provide an integrated diagnosis of sleep and neuropsychiatric findings. This approach enables a detailed characterization of sleep and neuropsychiatric manifestations in PCD patients.

In this cross-sectional study, we enrolled patients aged 5 to 60 years with a confirmed molecular diagnosis of PCD (*RSPH4A*, c.921+3_921+6delAAGT). A total of 15 non-related individuals—6 pediatric and 9 adults—were invited to take part in the evaluation of neuropsychiatric disorders and sleep-related manifestations, parents were not included since they did not have a PCD diagnosis. All patients were recruited from the accredited Puerto Rico PCD Center, and individuals with other rare genetic conditions (e.g., cystic fibrosis or immunodeficiencies) were excluded. Diagnosis was established in accordance with American Thoracic Society and PCD Foundation guidelines and was submitted and approved by the Ponce Medical School Foundation (PMSF) Institutional Board review (IRB) (See Figure 1) [14].

### 2.2. Clinical Assessment

Demographic and clinical data were collected during the evaluation, including age, gender, race, height, weight, body mass index (BMI), and BMI percentile for age. Neck circumference, a history of snoring, and any previously diagnosed neurobehavioral or psychiatric conditions (e.g., attention deficit hyperactivity disorder, oppositional defiant disorder) were also recorded. In addition, an ear, nose, and throat (ENT) examination was conducted to assess tonsil size using Brodsky grading for adenoidal hypertrophy, the presence of nasal polyps, Mallampati score, nasal swelling, and any evidence or history of chronic sinusitis.

### 2.3. Neuropsychiatric Assessment

Each patient underwent a comprehensive evaluation of their psychiatric and neuropsychiatric history through a structured interview. This process explored a range of self-reported symptoms, including difficulties with concentration, irritability, oppositional behaviors, hyperactivity, fatigue, restless sleep, anxiety, and depression. In addition, a mental status examination was conducted to assess current neuropsychiatric functioning. To further quantify mood- and anxiety-related symptoms, patients completed two standardized screening tools: the Patient Health Questionnaire-9 (PHQ-9) to assess depressive symptoms and the Generalized Anxiety Disorder-7 (GAD-7) to gauge anxiety levels. The results from these instruments guided subsequent evaluations and informed clinical decision-making [15].

### 2.4. Sleep Assessment

Prior to undergoing an attended Type I polysomnography, all patients completed standardized sleep-related questionnaires. For adults, the STOP-Bang and the Epworth Sleepiness Scale (ESS) were administered to evaluate the risk of obstructive sleep apnea (OSA) and excessive daytime sleepiness, respectively [16,17]. Pediatric patients completed the Pediatric Modified (PM) STOP-Bang and the Children and Adolescent Epworth Sleepiness Scale (ESS-CHAD), adapted tools designed to assess sleep-related breathing disorders and daytime sleepiness in younger populations [18,19]. In addition to these screening instruments, each patient was interviewed about specific sleep-related manifestations, such as snoring, limb movements, restlessness, sleepwalking, and sleep-talking.

### 2.5. Polysomnography Testing

Patients underwent polysomnography (PSG) testing using the Neurovirtual BWIII PSG polysomnogram, Ft Lauderdale, FL, USA. PSG was performed following the recommended American Academy of Sleep Medicine (AASM) standards including all general parameters (EEG, EOG, chin EMG, legs EMG, airflow signals, respiratory effort signals, oxygen saturation, body position, electrocardiogram, synchronized PSG video), montage and electrodes/sensors placement [19]. In addition, capnography was added to every study using end-tidal CO2 (ETCO2) sensors, Nonin RespSense Capnography monitor, Plymouth, MN, USA. The AASM recommended sample rates and filters settings were used from the AASM Scoring Manual, version 3. Data collected included total sleep time, time spent in every sleep stage (N1, N2, N3, R), percentage of time spent in each sleep stage, total recording time, sleep latency, wake after sleep onset (WASO), sleep efficiency, periodic limb movements in sleep (PLMS) reported as periodic limb movements index (PLMI), PLMS arousal index. Respiratory events included hypopneas and apneas (obstructive, central, and mix) which were reported using the Apnea–Hypopnea Index (AHI). The AASM recommended rule of hypopneas were used. Briefly, hypopneas were defined as a decrease in the peak signal excursion drop by >30% compared to baseline using he nasal pressure, duration of event of at least 10 s, and an associated consequence including either a 3% or greater oxygen desaturation from pre-event baseline; or when the event was associated with an arousal. An apnea was defined when a drop of >90% peak signal excursion using an oronasal thermal sensor (or airflow), and the duration of the 90% drop last 10 s or more. An apnea is considered obstructive if it is associated with inspiratory effort during the complete event, central if there is absence of inspiratory effort throughout the entire event and mix if it has an absence of inspiratory effort during the initial portion of the event followed by resumption of effort in the second portion of the event [20].

A sleep technician working under the supervision of a certified sleep technologist [Registered Sleep Technologist (RST certification)] performed the sleep studies. All studies were scored and analyzed by an independent board-certified sleep physician, who was blinded to patient evaluations and patient’s history.

Table 1 shows the AHI used to determine the presence and severity of OSA based on American AASM criteria. In adults, an AHI of 0–4.9 events per hour (events/h) was considered normal, 5–14.9 events/h was classified as mild, 15–29.9 events/h as moderate, and ≥30 events/h as severe. In children, an AHI of 0–0.9 events/h was deemed normal, 1.0–4.9 events/h was classified as mild, 5–9.9 events/h as moderate, and ≥10 events/h as severe (See Table 1) [20].

### 2.6. Statistical Methods

Descriptive statistics were utilized to summarize demographic and clinical characteristics, with continuous variables presented as medians and interquartile ranges (IQRs) and categorical variables as counts and percentages. Group comparisons between pediatric and adult patients were conducted using the Mann–Whitney U test for non-parametric continuous variables and paired t-test for significant differences between groups. To assess the effect size of group differences, Cohen’s d was calculated for continuous variables where applicable. A significance level of *p* < 0.05 was considered statistically significant; however, effect size was calculated to indicate the magnitude or practical importance of the result, specifically the extent of the observed effect, irrespective of sample size. All analyses were performed using GraphPad Prism version 10.4.1 (532) for Mac OS 15.3.

### 2.7. GenAI Use

In the preparation of this manuscript, the AI-assisting writing tool Paperpal (version 2.169.2) was used to refine language, improve readability, and ensure grammatical accuracy, the tool was not employed for generating original content, analyzing data, or formulating conclusions.

## 3. Results

### 3.1. Subject Characteristics and Demographics

Table 2 presents data on a total of 15 patients evaluated, comprising six pediatric patients (5–17 years of age) and nine adults (18–60 years of age). The median age for pediatric patients was 12.5 years (interquartile range [IQR]: 10.3–14.8), while for adults, it was 41 years (IQR: 25–55). All patients were of Hispanic ethnicity, *RSPH4A* homozygous, with a history of bronchiectasis, chronic sinusitis, and nNo < 77 nL/min. However, only two pediatric and two adult patients had a documented psychiatric history prior to the study.

### 3.2. Neuropsychiatric and Sleep Disorder Symptoms

Within the cohort, 67% of patients—both children and adults—experienced anxiety and/or depressive symptoms, ranging from mild to severe.

#### 3.2.1. Anxiety Symptoms

Among pediatric patients, those with OSA had a median GAD-7 score of 13 (IQR: 10.5–15.8), indicating moderate anxiety. By contrast, pediatric patients without OSA had a median GAD-7 score of 2 (IQR: 1–3), suggesting no clinically significant anxiety. In adults, 67% demonstrated anxiety symptoms irrespective of OSA status. Specifically, adults with OSA recorded a median GAD-7 of 7 (IQR: 4.5–8.5), while those without OSA had a GAD-7 of 6.5 (IQR: 4.5–10).

#### 3.2.2. Depressive Symptoms 

Depressive symptoms were noted in 83% of patients with OSA. Among pediatric patients with OSA, the median PHQ-9 score was 9 (IQR: 6.75–11.5), indicating mild-to-moderate depression. Pediatric patients without OSA had a lower median PHQ-9 of 5 (IQR: 2.5–7.5), reflecting minimal depressive symptoms. Adults with OSA showed mild depressive symptoms (PHQ-9: 6, IQR: 4–7.5), whereas adults without OSA exhibited scores suggestive of mild-to-no depression (PHQ-9: 4.5, IQR: 1.5–11.5).

#### 3.2.3. Sleep-Related Screening Measures 

**Figure 2:**
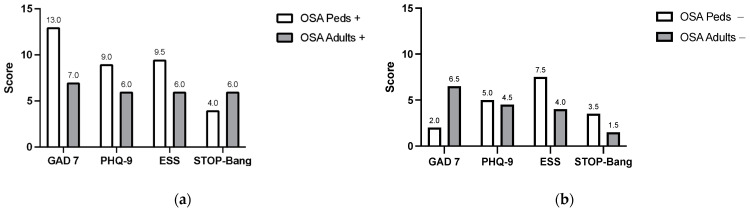
Neuropsychiatric and sleep questionnaires. Comparison of neuropsychiatric and sleep questionnaire scores among pediatric and adult patients with PCD, stratified by the presence or absence of OSA. The panel on the left (**a**) displays median scores for patients with OSA, while the panel on the right (**b**) shows scores for those without OSA. Neuropsychiatric measures include the Generalized Anxiety Disorder-7 (GAD-7) and Patient Health Questionnaire-9 (PHQ-9) for anxiety and depression, respectively. Sleepiness and OSA risk were assessed using the Epworth Sleepiness Scale (ESS) and STOP-Bang for adults, and the ESS-CHAD and PM-STOP-Bang for pediatric patients.

Figure 2 shows in the pediatric group, the overall STOP-Bang score was 4 (IQR: 3.5–4). Among those with OSA, the ESS-CHAD was 9.5 (IQR: 5.5–13.5) and the PM-STOP-Bang was 4 (IQR: 3.75–4.25). In comparison, pediatric patients without OSA had an ESS-CHAD of 7.5 (IQR: 6.25–8.75) and a PM-STOP-Bang of 3.5 (IQR: 3.25–3.75). 

For adults, the overall STOP-Bang was 4 (IQR: 1–4) and the ESS was 5 (IQR: 4–6). Adults with OSA had a median ESS of 6 (IQR: 6–8.5) and a STOP-Bang of 6 (IQR: 5–6), while adults without OSA had a lower ESS of 4 (IQR: 3.25–4.75) and a STOP-Bang of 1.5 (IQR: 0.25–2.75).

### 3.3. Polysomnography

#### 3.3.1. Sleep Architecture and Respiratory Values

In this cohort, 80% of patients (12 out of 15) demonstrated at least one sleep-related disorder. Among children, the median total sleep time was 400.5 min (IQR: 348.5–425.5), with a median arousal index of seven arousals per hour (IQR: 5.8–8.2) and a median sleep efficiency of 94% (IQR: 92.2–96.1). In adults, the median total sleep time was 351 min (IQR: 278–378), the median arousal index was 11.8 arousals per hour (IQR: 9–25.3), and the median sleep efficiency was 84.4% (IQR: 63.3–87.3). One patient exhibited alpha-delta sleep, indicating a possible underlying sleep disturbance.

Table 3 reflects respiratory assessments with a median AHI of 1.1 events/h (IQR: 1.0–1.3) in children and 1.3 events/h (IQR: 0.9–8.0) in adults. While the difference between groups was statistically significant in children (*p* = 0.03) and adults (0.007), the effect size was minimal (d = 0.03), suggesting limited clinical significance. Utilizing AASM criteria, four out of six (67%) of pediatric and three out of nine (33%) of adult patients met the criteria for OSA.

Time spent with oxygen saturation below 90% (T < 90%) was 0.05 min (IQR: 0–1.9) in children and 0.2 min (IQR: 0–3.5) in adults. This difference was not statistically significant in children (*p* = 0.36) or adults (*p* = 0.29) and demonstrated a negligible effect size (d = 0.001).

The median AHI in OSA-positive patients was significantly higher in adults (9.6, IQR: 8.8–10.4), but was not statistically significant (*p* = 0.16) compared to children (1.25, IQR: 1.1–1.75), in whom it was statistically significant (*p* = 0.03) with an effect size of d = 2.41, indicating a substantial difference between the two groups.

Sleep efficiency was significantly reduced in adults (84.4%, IQR: 63.3–87.3) (*p* ≤ 0.0001) compared to children (94%, IQR: 92.2–96.1) (*p* ≤ 0.0001). The effect size for this difference was d = 0.38, indicating a moderate effect.

The Arousal Index was significantly higher in adults (11.8 arousals/h, IQR: 9.0–25.3) (*p* = 0.001) compared to pediatric patients (7.0 arousals/h, IQR: 5.8–8.2) (*p* = 0.002), with a moderate effect size (d = 0.39).

Periodic Limb Movements Index exhibited no significant difference between groups, with both populations demonstrating a median of 0 leg movements per hour (*p* = 0.33 in children, *p* = 0.16 in adults).

Overall, patients primarily presented with mild-to-moderate OSA. All pediatric cases were classified as mild, while one of the three adult OSA cases reached moderate severity. Periodic Limb Movement Index (PLMI) > 15 was observed in one pediatric and one adult patient with OSA, as well as in one adult patient who did not meet the criteria for OSA.

#### 3.3.2. Movement and Other Sleep-Related Disorders

Sleep-related movement disorders were common among our patients. The most frequently reported condition was sleep-related bruxism, affecting 40% of the cohort. Of these cases, three (50%) were pediatric patients and two (22%) were adults. However, only one patient demonstrated bruxism corroborated by audio recordings during PSG.

Periodic limb movements in sleep (PLMS) were the second most common finding, present in 20% of patients. The median PLMI for both children and adults was 0 events/h (IQR: 0–1/h). Among pediatric patients, one out of six (17%) had a PLMI greater than five events/h, whereas two out of nine adults (22%) exceeded this threshold, including one with PLMI > 15 events/h. Additional isolated movement phenomena, such as hypnagogic foot tremors and rhythmic movement disorders, were observed in adult patients.

**Figure 3:**
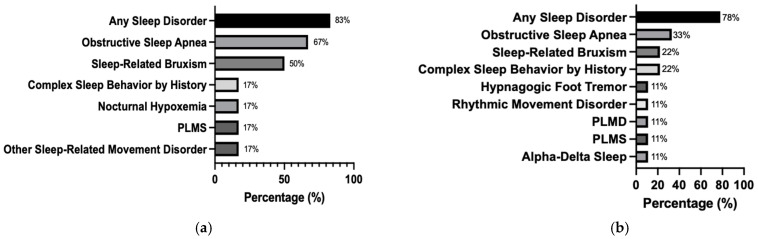
Sleep findings and disorders in pediatric (**a**) and adult (**b**) patients with PCD. The bar graphs represent the proportion of each group experiencing various sleep-related conditions, including OSA, sleep-related bruxism, complex sleep behaviors, nocturnal hypoxemia, PLMS, and other sleep-related movement disorders. The pediatric cohort (**a**) demonstrated a higher overall frequency of sleep disorders, particularly OSA and sleep-related bruxism, while the adult cohort (**b**) showed a more diverse range of sleep disturbances such as hypnagogic foot tremor, rhythmic movement disorder, PLMD, PLMS, and alpha-delta sleep.

Complex sleep behaviors (e.g., sleep talking, sleepwalking, confusional arousals, and dream-enactment behaviors) were either observed or self-reported by 20% of patients overall. Specifically, one pediatric patient (17%) and two adults (22%) exhibited these behaviors. However, no clear evidence of parasomnias was confirmed by PSG (See Figure 3).

## 4. Discussion

PCD is classically associated with hallmark respiratory complications, including chronic infections and bronchiectasis [2]. To date, no published studies have specifically examined the spectrum of sleep-related disorders and their corresponding neuropsychiatric manifestations in patients carrying the *RSPH4A* (c.921+3_921+6delAAGT) founder mutation. The data presented here are the first to highlight the presence of a wide range of sleep disturbances—encompassing sleep-disordered breathing, sleep-related movements, and complex sleep behaviors—alongside neuropsychiatric comorbidities in this genetically defined subset of the PCD population.

### 4.1. Neuropsychiatric Manifestations in PCD

Our investigation revealed that PCD, which is conventionally associated with respiratory complications, also exerts a substantial impact on neuropsychiatric health. The patient cohort exhibited a diverse array of symptoms including anxiety, depression, irritability, cognitive impairment, and diminished academic performance. These manifestations were observed across all age groups but demonstrated distinct patterns in pediatric and adult populations (see Appendix C).

All pediatric patients diagnosed with OSA exhibited moderate anxiety disorder (CI: 2.0–18.0), whereas those without OSA did not display such symptoms (CI: 0.0–13.0) (see Table A3). This finding indicates that sleep-disordered breathing (SDB) may either intensify or uncover underlying neuropsychiatric vulnerabilities in children, possibly due to the effects of chronic hypoxemia, altered sleep architecture, and psychological strain associated with chronic conditions. In the adult population, anxiety disorders were prevalent irrespective of OSA status (CI: 2.0–10.0 for OSA-positive adults and CI: 3.0–13.0 for OSA-negative adults), which may be attributed to the long-term accumulation of disease-related stress. Notably, depressive symptomatology was markedly more pronounced in adults with OSA (CI: 2–9) than in those without OSA (CI: 0–20), further supporting the well-documented relationship between OSA and affective disorders.

In contrast to previous studies that reported mild-to-moderate anxiety and depression in the broader PCD population (e.g., Graziano et al. [21]), our findings indicate that OSA may exacerbate these neuropsychiatric symptoms, particularly in adults. While studies such as Chang et al. [22] and Lee et al. [23] demonstrate a strong correlation between sleep apnea and mood disorders, our study highlights the importance of early recognition of sleep-disordered breathing as a potential contributor to the psychological burden in PCD patients.

While our study has demonstrated evidence for the presence of neuropsychiatric symptoms such as anxiety and depression in individuals with OSA, additional factors warrant further investigation and consideration [4,24,25]. Research has shown that obstructive sleep apnea (OSA) patients may experience disrupted sleep patterns and alterations in sleep architecture [26,27]. However, further investigation is necessary to elucidate other potential causes or implications regarding the function of primary ciliary dyskinesia (PCD) in the brain. This is due to the fact that PCD affects the primary cilia and could potentially influence the signaling of neuropsychiatric symptoms through mechanisms that are not yet fully elucidated [28,29].

Therefore, it is imperative to acknowledge that these conditions may share common underlying mechanisms, or at minimum, should serve as a model for investigating their connection to the brain. This approach is analogous to the extensive research conducted on sleep disorders such as narcolepsy, which has elucidated links between the hypocretin system, neuropsychiatric disorders, and sleep-related issues [30]. Moreover, research has demonstrated that disrupted neurodevelopmental signaling pathways, including Sonic Hedgehog (Shh) and Wnt, which are regulated by primary cilia, have been associated with other ciliopathies such as Bardet–Biedl syndrome and Joubert syndrome [6,28]. However, our study did not yield any conclusive findings, emphasizing the necessity for further research to elucidate the potential mechanism involved.

In addition, during their clinical interview, although most patients reported or exhibited a neuropsychiatric presentation, only five out of the fifteen patients reported seeking mental health treatment. While the total number of coping mechanisms for mental health was not quantified, the majority of patients reported emotional and social support as their primary coping mechanism, along with positive reinterpretation, growth, and acceptance. Nevertheless, research has demonstrated that patients may benefit from interventions that promote adaptive coping techniques, not only to evaluate current non-pharmacological treatment strategies but also to enhance quality of life in relation to their chronic medical condition [31].

The etiology of the presentation, whether it is a primary PCD manifestation or secondary to chronic disease burden, remains undetermined and necessitates further investigation. The underlying mechanisms linking PCD, sleep disturbances, and neuropsychiatric symptoms are currently hypothetical and require additional research [21,24,32]. While causality cannot be established, it is imperative to explore whether analogous mechanisms might influence the neuropsychiatric outcomes observed in PCD. Alternatively, chronic inflammation, intermittent hypoxemia, and disrupted neurotransmitter homeostasis—phenomena common in sleep apnea and chronic respiratory disease—could potentially contribute to these findings [33,34,35]. However, the extent to which these factors directly impact brain function in PCD remains to be elucidated.

Given the high prevalence of anxiety and depression in sleep disorders, integrating validated psychological screening tools into future studies on PCD-associated sleep dysfunction would provide a more comprehensive assessment of psychiatric burden and clinical relevance. Psychiatric symptoms in sleep disorders are frequently underrecognized; however, they significantly impact disease management, treatment adherence, and overall quality of life [36]. Standardized screening tools can facilitate the delineation of the severity and nature of neuropsychiatric symptoms, thereby enabling early intervention and tailored therapeutic strategies [21,36].

### 4.2. Sleep Respiratory Findings, and Prevalence

The ESS and STOP-Bang questionnaires proved effective in distinguishing individuals with and without OSA within the PCD cohort. Pediatric patients with OSA exhibited significantly higher ESS-CHAD scores (median: 9.5, IQR: 5.5–13.5) and PM-STOP-Bang scores (median: 4, IQR: 3.75–4.25) compared to their OSA-negative counterparts (ESS-CHAD: 7.5, IQR: 6.25–8.75; PM-STOP-Bang: 3.5, IQR: 3.25–3.75). Similarly, in adults, individuals with OSA demonstrated elevated ESS scores (median: 6, IQR: 6–8.5) and STOP-Bang scores (median: 6, IQR: 5–6), compared to ESS scores of 4 (IQR: 3.25–4.75) and STOP-Bang scores of 1.5 (IQR: 0.25–2.75) in OSA-negative adults. These findings highlight the utility of these screening tools in identifying sleep-disordered breathing, particularly in PCD populations, facilitating early diagnosis and intervention.

In the context of OSA prevalence, this study revealed a significantly higher prevalence of OSA among PCD patients compared to the general population. Senaratna et al. reported OSA rates of 9–38% in adults, with a higher prevalence among males [37]. According to the AASM criteria, OSA affects approximately 9% of women and 14% of men, defined as an apnea-hypopnea index (AHI) ≥ 5 events/h accompanied by clinical symptoms [37]. Among pediatric populations, OSA prevalence is typically 1–5%, although prevalence data using CMS criteria remain sparse [26].

In the present study, the overall prevalence of OSA in PCD patients was 46.7%, based on type I PSG. Prevalence rates were notably higher in pediatric patients (67%) compared to adults (33%). These findings align with those reported by Oktem et al., who identified OSA in 52% of pediatric PCD patients aged 6 months to 24 years, and Şişmanlar et al., who reported a prevalence of 60% among children aged 8–18 years using home sleep apnea testing (HSAT) [3,4]. Santamaria et al. reported an even higher prevalence of 100% in their PCD cohort, with severity classified as mild (19%), moderate (50%), and severe (31%) [2].

### 4.3. Anatomical and Physiological Contributors to OSA in PCD

The elevated prevalence of OSA in PCD patients appears to be attributable to disease-specific anatomical and physiological abnormalities. In this study, pediatric patients with OSA exhibited higher tonsil grades (median: 3, IQR: 2.5–3.5), suggesting that adenotonsillar hypertrophy is a significant contributor to airway obstruction (See Table A1). Adult patients demonstrated elevated Mallampati scores (median: 3, IQR: 2–3), indicative of upper-airway narrowing exacerbated by chronic inflammation and tissue remodeling (See Table A2). These structural abnormalities, which are common in PCD, reflect persistent upper airway inflammation and edema [5]. Furthermore, nasal polyps and sinusitis, which are frequently observed in PCD, contribute to increased upper airway resistance. Impaired mucociliary clearance, in conjunction with chronic infection and inflammation, creates a cycle of obstruction and hypoxemia, perpetuating the development of OSA [2,3].

### 4.4. Impact of OSA on Pulmonary Health

OSA and PCD exhibit a bidirectional relationship. Untreated OSA exacerbates pulmonary dysfunction, accelerates bronchiectasis, and exacerbates respiratory symptoms. Recurrent hypoxemia and hypercapnia, characteristic features of OSA, impair mucociliary clearance and increase secretion retention, leading to chronic infection. This cycle induces systemic inflammation, vascular strain, and endothelial dysfunction, further compromising pulmonary health [33,34,38]. In our cohort, one of our patients exhibited nocturnal hypoxemia unrelated to respiratory events. This finding suggests potential alternative mechanisms, such as ventilation–perfusion mismatch resulting from chronic pulmonary infections or impaired central respiratory control. These complexities underscore the necessity for further research to elucidate the nonobstructive causes of hypoxemia in patients with PCD.

### 4.5. Gender and Age-Related Patterns

No significant sex differences in OSA prevalence were identified in this study (*p* = 0.3), although the small sample size may have limited these observations. Among the pediatric patients, OSA was equally distributed, with 66.7% affected in both males and females. In adults, OSA prevalence was slightly higher in males (33.3%) than females (20%), mirroring trends in the general population. The severity of OSA varied according to the age group. Pediatric cases were predominantly mild, whereas adult cases tended to have moderate-to-severe presentations. This progression likely reflects cumulative airway remodeling, chronic inflammation, and the emergence of age-related comorbidities [27,35,39].

### 4.6. Clinical Implications and Management Strategies

The high prevalence of OSA in PCD underscores the need for routine screening and early intervention to mitigate the impact of OSA on pulmonary and neuropsychiatric outcomes [2,8,37]. Validated screening tools such as ESS-CHAD, STOP-Bang, and PM-STOP-Bang effectively identified high-risk individuals in this cohort, supporting their implementation in standard care protocols for patients [37]. Management strategies must address both the anatomical abnormalities and physiological contributors to airway obstruction. Continuous positive airway pressure (CPAP) therapy remains the primary intervention for treating moderate-to-severe OSA and improves oxygenation, ventilation, and sleep quality [2,3]. Surgical interventions, including adenotonsillectomy or polypectomy, may be necessary for patients with significant anatomical obstruction, offering reductions in upper airway resistance and an enhanced quality of life [3,4]. Supplemental oxygen therapy or ventilatory support may be indicated for individuals with nocturnal hypoxemia unrelated to obstructive events, emphasizing the importance of individualized care [4,37].

### 4.7. Sleep-Related Movement Disorders and Complex Sleep Behaviors

Prevalence and manifestations in PCD sleep-related movement disorders exhibited a notable prevalence in our cohort of patients with PCD carrying the *RSPH4A* (c.921+3_921+6delAAGT) mutation. The most frequently observed manifestation was sleep-related bruxism, affecting 40% of the patients. Among those affected, 50% were pediatric patients and 33% were adults. These rates significantly exceed the general population estimates, where bruxism affects 6.96–11.30% of children and 11.14–18.64% of adults [40]. Diagnoses were primarily based on clinical history, with only one case confirmed by PSG using EEG and sound correlation. Notably, bruxism is strongly associated with anxiety and OSA, as patients display elevated GAD-7 and PHQ-9 scores, suggesting a psychological component linked to both conditions [25]. PLMS was the second most prevalent sleep-related movement disorder, affecting 20% of the patients. This exceeds the general population prevalence of 4–11% [41]. Among the affected individuals, two adults and one pediatric patient exhibited PLMS in association with sleep-disordered breathing (SDB)/OSA. The pediatric case was particularly noteworthy for predominant PLMS during REM sleep, an atypical presentation that warrants further investigation [42,43]. In addition to isolated conditions, several patients demonstrated multiple sleep-related movement disorders, including hypnagogic foot tremors, rhythmic movement disorders, and alpha-delta sleep patterns. One adult patient exhibited large-amplitude leg movements consistent with rhythmic movement disorder along with alpha-delta sleep. Notably, PSG findings in this patient revealed no evidence of associated RLS or OSA, suggesting that these disorders warrant further investigation to explore potential relationships with ciliary dysfunction or other PCD-specific mechanisms affecting the brain.

Complex sleep behaviors, including somniloquy, somnambulism, confusional arousals, and dream enactment, were observed in 20% of our patient population. Although these parasomnias were not definitively confirmed through PSG, they raise significant questions regarding the neurodevelopmental and physiological mechanisms underlying sleep disturbances in PCD. A comprehensive review of the extant literature revealed no prior reports of such PCD disorders, suggesting that they may represent previously unrecognized comorbidities associated with the *RSPH4A* founder mutation [32,33].

These complex sleep behaviors present a diagnostic challenge, as they may have diverse etiologies. While some patients clearly exhibited sleep-related movement disorders, others demonstrated behaviors that were not fully elucidated. This variability necessitates thorough evaluation, particularly in patients with a psychiatric history or those presenting with psychiatric symptoms but not currently undergoing treatment. In such cases, the observed symptoms may not correlate with their clinical presentation, potentially indicating a functional neurological disorder or conversion disorder. Consequently, the current presentation of these patients may not necessarily be attributed to a sleep disorder of neurological origin, but rather to a psychiatric manifestation. To differentiate between neurological and primarily psychiatric presentations, further research in a multidisciplinary care setting is essential [44,45,46].

### 4.8. Mechanisms Underlying Sleep Disturbances in PCD

The primary cilium plays a crucial role in neurodevelopmental signaling pathways, including Sonic Hedgehog (Shh) and Wnt pathways. Disruptions in these pathways have been associated with neuropsychiatric disorders, cognitive impairments, and sleep disturbances in ciliopathies such as Bardet–Biedl syndrome and Joubert syndrome [13,28,29]. While direct evidence linking PCD to neurodevelopmental sleep disorders is limited, it is plausible that primary ciliary dysfunction contributes to observed movement and behavioral disorders through impaired neuronal signaling. Chronic hypoxemia and systemic inflammation, both of which are associated with OSA in PCD, likely exacerbate sleep disturbances. Hypoxemia is known to impair neurotransmitter regulation and brain plasticity, potentially magnifying the neurological consequences of ciliary dysfunction [6,22,24]. This interplay between recurrent hypoxemia, inflammation, and disrupted ciliary signaling pathways may elucidate the spectrum of sleep-related disorders identified in this cohort.

### 4.9. Implications for Clinical Practice

Recognizing sleep-related movement disorders and complex sleep behaviors as under-diagnosed manifestations of PCD is essential for improving patient outcomes. These conditions can significantly impact the quality of life and may reflect broader neurodevelopmental or physiological disruptions related to ciliary dysfunction. Routine PSG evaluations should be considered for patients presenting with bruxism, PLMS, or parasomnias, to accurately characterize these disturbances and inform targeted interventions [47,48]. Our findings suggest that bruxism is strongly associated with anxiety and OSA in patients with PCD, emphasizing the importance of the comprehensive assessment and management of these interrelated conditions. For cases of PLMS or rhythmic movement disorders, PSG is indispensable for diagnostic accuracy and differentiation from other sleep disorders [33,47].

### 4.10. Future Research Directions

Further research is essential to elucidate the interplay between ciliary dysfunction, sleep-disordered breathing, pulmonary health, and its neuropsychiatric manifestations in patients with PCD. To comprehensively assess cognition in PCD-associated sleep disorders, a range of standardized neuropsychological tests should be incorporated in multiple cognitive domains. These include tests for attention and processing speed (i.e., trail-making test), executive function and cognitive flexibility (Wisconsin Card-Sorting Test [WCST]). Additionally, assessments of mood (Beck Depression Inventory-II [BDI-II]), sleep (Pittsburgh Sleep Quality Index [PSQI]), and the Neuropsychiatric Inventory (NPI) should be included, among others. These instruments, when integrated into PCD research, can help elucidate the impact of sleep dysfunction, hypoxia, and chronic disease burden on cognition and mental health [49,50,51,52].

Furthermore, subsequent investigations should examine the function of primary cilia in respiratory regulation and their influence on ventilatory drive during sleep, as well as their potential impact on both central and peripheral respiratory mechanisms [5,28]. Non-obstructive causes of nocturnal hypoxemia, such as ventilation–perfusion mismatch and neurocognitive factors, necessitate further exploration to elucidate diverse physiological impairments in patients with PCD [2,37]. Longitudinal studies assessing the efficacy of interventions such as CPAP therapy, adenotonsillectomy, polypectomy and sleep hygiene are essential to evaluate their long-term benefits in reducing disease burden and improving sleep quality [2,3,52]. Moreover, research on the molecular mechanisms underlying chronic airway inflammation and its role in sleep-related disorders may provide insights into targeted treatments aimed at mitigating the systemic and pulmonary consequences of PCD [4,5,6].

### 4.11. Limitations

While this study provides valuable insights into the neuropsychiatric and sleep-related manifestations of PCD, several limitations must be acknowledged. The small sample size and focus on a specific genetic mutation within a Puerto Rican cohort restricts the generalizability of the findings to broader populations. Furthermore, potential outliers and confounding variables, such as medications that could affect sleep and present with neuropsychiatric manifestations, along with other unanticipated confounding variables, should be considered. Additionally, technical limitations inherent in small-scale studies may have influenced the results. Cognitive and neuropsychiatric evaluations were constrained by funding limitations. Comprehensive neuropsychological assessments, which could have provided deeper insights into the cognitive and emotional impacts of PCD, have not been conducted. Instead, cognitive data were primarily derived from subjective reports during clinical interviews, which may have lacked precision. Psychosocial stressors, known to contribute to mood disorders, have not been fully explored, further limiting the ability of the study to contextualize neuropsychiatric outcomes [20,21,44].

In the realm of sleep-related findings, the protocol prioritized objectivity, but limited the assessment of certain conditions. For instance, circadian rhythm disorders, insomnia parameters, such as sleep latency and wake-after-sleep onset, and specific aspects of complex sleep behaviors have not been evaluated using standardized scales. This may have resulted in the underestimation of these conditions. The absence of a control group further constrained our ability to establish causative relationships between observed sleep disturbances and PCD. Moreover, the evaluation of sleep-related movement disorders and parasomnias relies heavily on clinical history, with limited polysomnographic confirmation, particularly for complex sleep behaviors [44,47,48].

Despite these limitations, this study serves as a critical foundation for future research in sleep and neuropsychiatric related disorders in PCD. Larger multicenter studies with diverse populations and control groups are essential to validate these findings and extend their applicability. The incorporation of objective neuropsychological assessments and standardized sleep measures will enhance our understanding of PCD’s impact on pulmonary, neuropsychiatric, and sleep health. Expanding these efforts will facilitate the development of targeted therapeutic strategies and improve outcomes in PCD [1,21,52].

## 5. Conclusions

Primary ciliary dyskinesia (PCD) is a rare genetic disorder primarily characterized by respiratory dysfunction; however, our findings suggest it may also be associated with sleep-related disturbances and neuropsychiatric symptoms, particularly in patients with PCD *RSPH4A* founder mutation. In this small and heterogeneous cohort, sleep disturbances such as obstructive sleep apnea (OSA) and sleep-related movement disorders were observed. However, due to the age diversity and sample limitations, further research is necessary to elucidate the extent and clinical significance of these findings.

Neuropsychiatric symptoms, including anxiety and depression, appeared to co-occur with sleep disturbances, though additional studies with larger, more representative samples are required to determine their relationship in PCD. Moreover, the interplay between psychiatric manifestations and potential organic causes remains unclear. Some patients exhibited complex sleep behaviors that were not fully elucidated as to whether they represent underlying functional neurological disorders or genuine neurologic manifestations of the disease itself. Differentiating between neurological and psychiatric etiologies in these cases is critical and warrants further investigation in a multidisciplinary setting.

While interventions such as CPAP therapy and other therapeutic strategies may ameliorate symptoms, their long-term efficacy in this population remains to be fully explored. Future studies should aim to incorporate larger cohorts, standardized assessments, and effect size calculations to better elucidate the impact of sleep disturbances and neuropsychiatric complications in PCD [53]. A multidisciplinary approach, integrating pulmonology, sleep medicine, and neuropsychiatry, may be essential for optimizing patient care and improving diagnostic precision.

## Figures and Tables

**Table 1:** AHI values used for diagnosis for obstructive sleep apnea in children and adult patients upon polysomnography testing according to the AASM.

Severity	Adults (AHI Events/h)	Children (AHI Events/h)
Normal	0–4.9	0.0–0.9
Mild	5–14.9	1.0–4.9
Moderate	15–29.9	5–9.9
Severe	≥30	≥10

Note: AHI = Apnea–Hypopnea Index; events/h; AASM = American Academy of Sleep Medicine.

**Table 2:** Demographics and clinical history observed in the PCD *RSPH4A*, c.921+3921+6delAAGT population in both the adult and pediatric Hispanic population in Puerto Rico.

Clinical Characteristics (n = 15)	Pediatric (n = 6)	Adults (n = 9)
Female Patients	3	6
Male Patients	3	3
Hispanic Ethnicity	6	9
*RSPH4A* Homozygosity	6	9
Bronchiectasis	6	9
Chronic Sinusitis	6	9
nNO Levels below 77 nL/min	6	9
Psychiatric History	2	2

**Table 3:** Overnight PSG scores in pediatric and adult populations with PCD.

	Pediatric	*p* Value	Adults	*p* Value	Effect Size
AHI (Events/h)	1.1 (IQR: 1–1.3)	0.03	1.3 (IQR: 0.9–8)	0.007	0.03
T < 90 Saturation (min)	0.05 (IQR: 0–1.9)	0.36	0.2 (IQR: 0–3.5)	0.29	0.001
OSA Severity		0.03			
❖ Mild	1.25 (IQR: 1.1–1.75)		9.6 (IQR: 8.8–10.4)	0.16	2.41
❖ Moderate	**		18.1 (IQR: 18.1)	0.47	***
❖ Severe	**		**		
Sleep Efficiency (%)	94 (IQR: 92.2–96.1)	<0.0001	84.4 (IQR: 63.3–87.3)	<0.0001	0.38
Arousal Index (Arousals/h)	7 (IQR: 5.8–8.2)	0.002	11.8 (IQR: 9–25.3)	0.001	0.39
PLMS Index (LMs ^†^/h)	0 (IQR: 0–0.9)	0.33	0 (IQR: 0–1)	0.16	0

**Note:** IQR denotes interquartile range; ** Did not met criteria for OSA severity; *** Could not be performed due to lack of data; ^†^ LMs/h stands for leg movements per hour.

## Data Availability

The original contributions presented in this study are included in the article. Further inquiries can be directed to the corresponding author.

## Abbreviations

The following abbreviations are used in this manuscript:

OSA	Obstructive Sleep Apnea
PCD	Primary Ciliary Dyskinesia
PLMS	Periodic Limb Movements of Sleep
CPAP	Continuous Positive Airway Pressure
PSG	Polysomnography
ESS	Epworth Sleepiness Scale
ESS-CHAD	Epworth Sleepiness Scale for Child and Adolescents

## Appendix A

**Table A1:** Sleep scales and other physical examination in pediatric patients.

Pediatric Patient	BMI (Percentile)	Neck Circumference	Tonsil Size	Mallampati Score	ESS-CHAD	PM-STOP-Bang
1	21.7 (99.5th)	11.0	4	2	6	5
2	14.0 (6th)	10.0	1	1	5	4
3	21.5 (86th)	12.5	0	2	13	4
4	17.2 (19th)	11.5	1	1	10	3
5	22.3 (79th)	11.5	4	2	4	3
6	25.8 (87th)	14.0	2	4	15	4

## Appendix B

**Table A2:** Sleep Scales and Other Physical Examination in Adult Patients.

Adult Patient	BMI	Neck Circumference	Tonsil Size	Mallampati Score	ESS	STOP-Bang
1	21.9	11.5	1	2	12	1
2	20.1	11.5	1	1	4	2
3	21.6	11.0	1	4	3	1
4	31.9	13.0	1	1	0	0
5	29.8	18.0	1	4	6	6
6	30.7	14.0	1	3	4	4
7	32.3	14.0	1	3	11	6
8	20.7	11.0	1	3	6	4
9	18.2	14.5	1	2	5	4

## Appendix C

**Table A3:** Neuropsychiatric Manifestations in Pediatric and Adult Patients with PCD.

Neuropsychiatric Symptoms	Total	Pediatric	Adult
Anxiety (By GAD-7)	9	9.7	6.5
Depression (By PHQ-9)	9	6.7	7.5
Restless Sleep (%)	10	67	67
Concentration (%)	11	83	67
Hyperactivity (%)	6	50	33
Irritability (%)	14	100	89
Oppositional Behavior (%)	3	50	0
Poor Academic Performance (%)	4	50	11
